# Tumour reoxygenation after intratumoural hydrogen peroxide (KORTUC) injection: a novel approach to enhance radiosensitivity

**DOI:** 10.1038/s44276-024-00098-y

**Published:** 2024-10-08

**Authors:** Samantha Nimalasena, Selvakumar Anbalagan, Carol Box, Sheng Yu, Jessica K. R. Boult, Nigel Bush, Louise Howell, Victoria Sinnett, William Murphy, John Yarnold, Simon P. Robinson, Navita Somaiah

**Affiliations:** 1 Division of Radiotherapy & Imaging, The Institute of Cancer Research, London, UK; 2 The Royal Marsden NHS Foundation Trust, London, UK; 3 Core Research Facilities, The Institute of Cancer Research, London, UK; 4 Biological Service Unit, The Institute of Cancer Research, London, UK

## Abstract

**Background:**

KORTUC (0.5% hydrogen peroxide (H_2_O_2_) in 1% sodium-hyaluronate) releases cytotoxic levels of H_2_O_2_ in tissues after intratumoural injection. High levels of tumour control after radiotherapy plus KORTUC are reported in breast cancer patients. Here, we use human xenograft models to test the hypothesis that oxygen microbubbles released post-KORTUC are effective in modifying the hypoxic tumour microenvironment.

**Methods and materials:**

Pimonidazole and Image-iT™ Red (live hypoxia marker) were utilised to assess dose-dependent changes in hypoxia post-H_2_O_2_ in HCT116 and LICR-LON-HN5 spheroids. Using a dual 2-nitroimidazole-marker technique and phospho-ATM we evaluated changes in hypoxia and reactive oxygen species (ROS) respectively, in HCT116 and LICR-LON-HN5 xenografts following intratumoural KORTUC.

**Results:**

A significant reduction in Image-iT™ Red fluorescence was observed in spheroids 1 h post-H_2_O_2_ at ≥1.2 mM, maintained at 24 h. Ultrasound demonstrated sustained release of oxygen microbubbles within tumours, 1 h post-KORTUC. Hypoxia markers demonstrated significant tissue reoxygenation in both models post-KORTUC and significantly increased phospho-ATM foci reflecting increased ROS production.

**Conclusion:**

Intratumoural KORTUC represents a novel oxygen delivery method, which can be exploited to enhance radiation response. If efficacy is confirmed in the ongoing phase 2 breast trial it could improve treatment of several tumour types where hypoxia is known to affect radiotherapy outcomes.

## Introduction

Pre-clinical and clinical studies have reported intratumoural administration of H_2_O_2_ as a potential radiosensitiser in solid tumours [1–4]. In vivo use was enabled by mixing H_2_O_2_ with 0.83% sodium hyaluronate gel to produce a 0.5% H_2_O_2_ solution (KORTUC) to ensure stability and minimise pain on injection [5]. Our Phase I clinical trial (NCT02757651) has confirmed safety and shown promising tumour responses in locally advanced breast cancer [2]. An enhanced cytotoxic effect and significant tumour growth delay was observed when H_2_O_2_ was combined with ionising radiation in radioresistant cell lines and in murine tumour models [5–7]. This effect was not only due to enhanced DNA damage as would be expected following radiation. Instead, this was demonstrated to be due to permeabilisation of the lysosomal membrane, with resultant release of heavy metal ions (e.g. Ferrous ion), disruption of the mitochondrial membrane, and apoptosis [8–10].

When KORTUC is injected into a tumour, H_2_O_2_ decomposes into water and oxygen, or in the presence of ferrous iron generates oxygen and OH• radicals. The former reaction is catalysed by intracellular catalase/peroxidases and the later by Fenton and Haber-Weiss reactions. The immediate formation of oxygen microbubbles within the tumour is visible under ultrasound imaging and used to guide drug delivery throughout the tumour volume [2]. This observation led us to investigate reoxygenation as an additional mechanism of radiosensitisation.

Tumour hypoxia is a well-established cause of radiation resistance, and strategies designed to increase oxygenation for radiotherapeutic gain are continually being sought following the seminal studies by Gray et al. in the 1950s [11–13]. Intratumoural administration of KORTUC may represent a novel means of oxygen delivery directly into the tumour. This study tests the hypothesis that tumour reoxygenation occurs following release of molecular oxygen, which can be exploited to enhance response to radiation.

## Materials and methods

### Cell culture

Human HCT116 colorectal (ATCC) and LICR-LON-HN5 (HN5) head and neck cancer cells (The Ludwig Institute for Cancer Research) were grown in Dulbecco’s modified eagle’s medium (ThermoFisher Scientific, UK) supplemented with 10% foetal bovine serum (Pan Biotech, UK) in a humidified incubator with 5% CO_2_. Cell lines were authenticated by short tandem repeat (STR) profiling and confirmed to be mycoplasma-free by PCR analysis (Surrey Diagnostics, UK).

### Drug preparation

Serial dilutions of hydrogen peroxide (#H1009, Sigma Aldrich, UK) were prepared in complete cell culture medium (0–9.6 mM). For intratumoural KORTUC injection, hydrogen peroxide (3% v/v, Stockport Pharmaceuticals, UK) was mixed with sodium hyaluronate gel (OSTENIL^®^, 20 mg sodium hyaluronate; AAH Pharmaceuticals, UK) and freshly prepared under sterile conditions to achieve a final clinical concentration of 150 mM (0.5% v/v).

### Spheroid experiments

Spheroids were formed by seeding 300 HCT116 or 2000 LICR-LON-HN5 cells per well of an ultra-low attachment 96-well plate. On Day 4, spheroids (400–700 μm diameter) were treated with H_2_O_2_ (0–9.6 mM). For immunohistochemical detection of hypoxia, spheroids were incubated with 200 µM pimonidazole (Hypoxyprobe™-1, HP-100, Hypoxyprobe, USA) for 24 h prior to fixation with 4% paraformaldehyde. Fixed spheroids were embedded in 2% agarose, dehydrated and embedded in paraffin. 5 µm sections were stained with haematoxylin and eosin (H&E), with adjacent sections exposed to anti-pimonidazole antibodies conjugated to FITC to detect bioreduced adducts, and DAPI to counterstain nuclei. H&E-stained sections were imaged on a Hamamatsu Nanozoomer-XR and fluorescent staining was imaged on a Zeiss Axioscan Z1.

For live hypoxia imaging, spheroids were incubated for 24 h with 5 µM Image-iT™ Red Hypoxia Reagent (ThermoFisher Scientific) and treated with the same range of H_2_O_2_ concentrations (0–9.6 mM). Brightfield and fluorescence images of spheroids were acquired, at intervals up to 24 h post-treatment, with a Celigo imaging cytometer (Nexcelom Bioscience, USA) using the ‘Tumoursphere 1’ application, and spheroid diameter and average fluorescence intensity quantified using Celigo software.

### Xenograft experiments

All animal studies were conducted in accordance with the local ethical review panel, the UK Animals (Scientific Procedures) Act 1986, the UK National Cancer Research Institute guidelines for the welfare of animals in cancer research and the ARRIVE (Animal Research: Reporting of In Vivo Experiments) guidelines [14, 15]. Six-week-old female *Foxn1* nude mice (Charles River Laboratories, UK) were injected with 3 ×10^6^ tumour cells in Hanks’ balanced salt solution subcutaneously on the right flank. Mice were housed in specific pathogen-free rooms in autoclaved, aseptic microisolator cages with a maximum of 5 animals per cage. Mice were allowed access to food and water *ad libitum*. Mice were treated when their tumour volume reached ~400 mm^3^ as measured using calipers and the ellipsoid formula (π/6 × d1 × d2 × d3). Mice were randomly assigned to three groups, Group 1: non-injected control, Group 2: vehicle-injected control (sodium hyaluronate alone), and Group 3: KORTUC (sodium hyaluronate + H_2_O_2_). For HCT116, Group 1: *n* = 3; Group 2: *n* = 3 and Group 3: *n* = 6 and for LICR-LON-HN5, Group 1: *n* = 8; Group 2: *n* = 8 and Group 3: *n* = 8. Xenograft experiments were not blinded.

For intratumoural KORTUC injections mice were anaesthetised with an intraperitoneal injection of 80 mg/kg ketamine (Zoetis UK Ltd) and 10 mg/kg xylazine (Elanco Europe Ltd, UK) in water. Ultrasound (US) guidance was used to ensure drug delivery within the tumour. This comprised a heated platform upon which anaesthetised mice were positioned, with an adjustable clamp to manoeuvre a 0.5 ml syringe fitted with a 21G needle. The height of the platform was adjusted such that the immobilised animal was brought into contact with the US probe (Toshiba Aplio XG PLT-1204BT linear array) prior to intratumoural injection.

### Dual hypoxia marker technique

A dual hypoxia marker staining technique was used to assess any H_2_O_2_-induced changes in tumour hypoxia as previously described [16]. In brief, the 2-nitroimidazole CCI-103F (Hypoxyprobe-F6, HP4-100, Hypoxyprobe, 80 mg/kg in 10% DMSO, 90% peanut oil) was injected intraperitoneally for determining baseline tumour hypoxia and allowed 2 h to undergo full bioreduction within hypoxic tumour regions (<10 mmHg). Subsequently, 0.05 ml of either KORTUC or vehicle was slowly injected intratumourally under US guidance, and the tumour continuously imaged with US for 1 h. Subsequently, a second 2-nitroimidazole, pimonidazole (60 mg/kg in PBS), was administered intraperitoneally and a further 45 min allowed for hypoxic bioreduction of this marker. Animals were then killed by cervical dislocation and tumours were rapidly excised, cryopreserved in liquid nitrogen and stored at −80 °C. Tumours were embedded in OCT compound and 10 μm frozen sections cut using a cryotome (−22 °C; Leica). Slides were stored at −20 °C until staining.

### Immunohistochemistry (IHC) and Immunofluorescence (IF) staining

IHC and IF staining was carried out as previously described [16]. Frozen sections were fixed in ice-cold acetone and rehydrated in PBS. Sections were blocked with 2% BSA and 5% goat serum for 1 h and incubated overnight with rabbit anti-CCI-103F antibodies (HP4-100 anti F6 Rabbit, 1:100) at 4 °C. The slides were incubated at room temperature for 90 min with TRITC-conjugated goat anti-rabbit secondary antibodies (AF594, #ab150084, Abcam,1:200). Sections were then stained for pimonidazole adducts using a FITC-conjugated anti-pimonidazole antibody (#HP2-100, mouse FITC-mAb, 1:200) for 2 h at room temperature. On separate sections, phospho-ATM (#sc-47739, clone: 10H11.E12, 1:200) was co-stained overnight along with CCI-103F at 4 °C and detected with secondary antibodies conjugated to AF488 (#ab150113, 1:200) and AF594 respectively. Slides were washed then stained with or without Hoechst 33342 (10 μg/ml, 15 min) and coverslips mounted with Vectashield Plus. An additional serial section from each xenograft was stained with H&E.

### Microscopy and Image analysis

Whole tissue sections were imaged using a motorised scanning stage (Prior Scientific Instruments, Cambridge, UK) attached to a BX51 microscope and DP74 camera and driven by cellSens Dimension v1.18 imaging software (Olympus Optical, UK), using a 4x objective, pimonidazole (λ_em_ 518 nm) and CCI-103F (λ_em_ 617 nm). Composite fluorescence images were stitched from individually acquired images. Images of immunofluorescence staining of phospho-ATM and CCI-103F were obtained from xenograft sections using a Zeiss LSM700 confocal microscope using a 63x objective.

Image analysis was performed using cellSens and Fiji, ImageJ application (v2.9.0/1.53 s) [17]. The entire tumour-bearing region was outlined as the region of interest (ROI), excluding any overlying skin and normal tissue for analysis [16]. A global preset intensity using adaptive threshold was implemented using cellSens to define and quantify hypoxia adduct formation. The difference between the hypoxia markers was expressed as a log2 fold change (Fc).

A further detailed image analysis was carried out using Fiji ImageJ. Images were converted into 8-bit, the OTSU threshold was implemented, and an appropriate threshold was determined taking into consideration the background and hypoxia negative region. Areas of reoxygenation were identified using Fiji image ‘subtraction’ and ‘AND’ arithmetic operators on the OTSU threshold implemented image (Supplementary Fig. S1). Based on areas of mismatch, three categories were defined: reoxygenated (CCI-103F ‘–’ overlapping regions), new hypoxia (Pimonidazole ‘–’ CCI-103F), no change (overlapping regions). For example, to estimate the extent of reoxygenation within individual tumours, a log_2_Fc was calculated using the integrated density of the region stained only for CCI-103F, and subtracting the overlap region between CCI-103F and pimonidazole (Supplementary Fig. S1). A log2Fc < 0 indicates absence of reoxygenation and a log2Fc > 1 is considered reoxygenation.

### Phospho-ATM foci quantification

Regions within the tumour that underwent reoxygenation were identified through comparison between the thresholded images obtained from dual hypoxia marker staining, ImageJ and the normalised image generated using ΔScore (Supplementary Fig. S2). Regions of reoxygenation common to all three images were identified, and compared with dual stained CCI-103F/phospho-ATM images, to identify corresponding regions. Six such regions per tumour section were chosen randomly and used for the analysis as shown in Supplementary Fig. S2, after manual QC to exclude any regions with artefacts. At least 5 high-power fields (63x) were imaged in each of the six regions identified above. A minimum of 50 cells were scored in each high-power field, to quantify nuclear phospho-ATM expression. Cells were scored for presence or absence of phospho-ATM, and further subdivided into those with >5 phospho-ATM foci, in both CCI-103F positive cells (hypoxic at baseline) and the surrounding regions with no uptake of CCI-103F (not hypoxic at baseline) (Supplementary Fig. S2). A box and whisker plot was generated to compare phospho-ATM expression in each of the 3 conditions (non-injected control, vehicle-injected control and KORTUC injected xenografts).

### Statistical analyses

Statistical analyses were performed using Prism v9.3.1 (GraphPad) and data are presented as mean ± standard deviation. Significant differences were identified using Student’s *t* test with a significance level of 5%. To determine the extent of tumour reoxygenation in xenograft studies, log2Fc (fold change) was calculated and log2Fc > 1 was defined as reoxygenated. To compare multiple group testing for phospho-ATM staining, a two-way ANOVA using Šídák multiple comparison was performed. ‘*’ *p* < 0.05, ‘**’ *p* < 0.001, ‘***’ *p* < 0.0001, ‘ns’ non-significant.

## Results

### H_2_O_2_ induced reoxygenation is dose and time dependent in 3D spheroid models

**Fig. 1:**
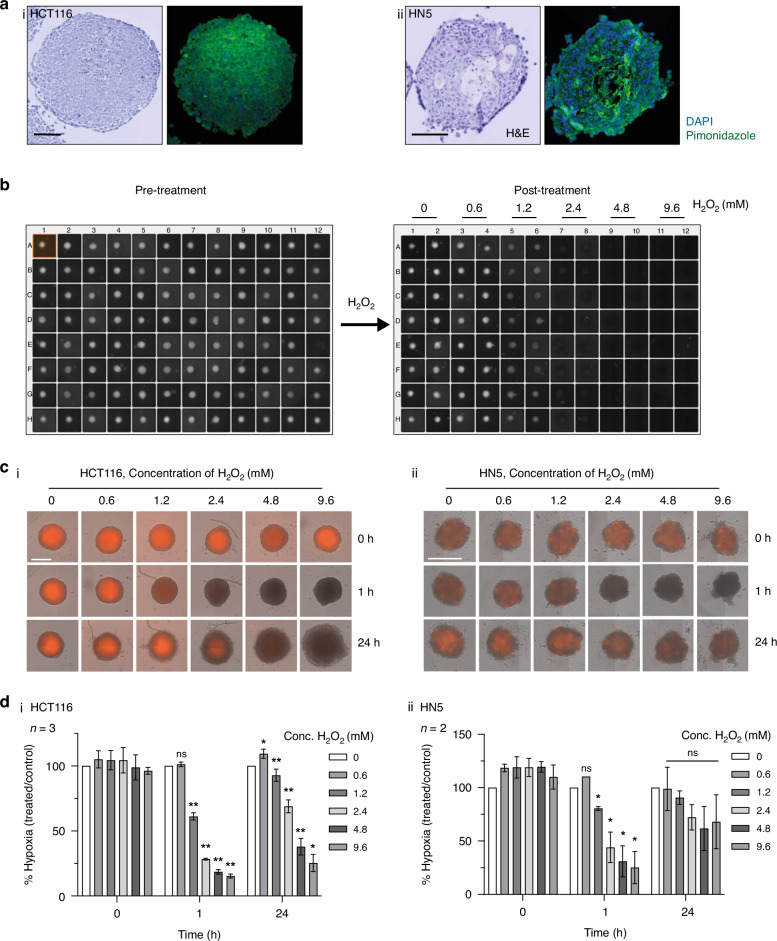
Dose dependent reoxygenation following hydrogen peroxide treatment of spheroids. **a** H&E and pimonidazole staining confirmed the presence of hypoxia in (i) HCT116 and (ii) HN5 spheroids. Scale bar = 250 µm. **b** Representative fluorescence image of a 96-well plate with HCT116 spheroids pre-treated with Image-IT™ Red (live cell hypoxia dye; baseline hypoxia) and images acquired 1 h post-treatment with H_2_O_2_ (0–9.6 mM). **c** Merged brightfield and fluorescence images of (i) HCT116 and (ii) HN5 spheroids pre-treated with Image-iT™ Red (24 h) and then treated with H_2_O_2_ (0–9.6 mM). Representative spheroids are shown. The apparent increase in size of HCT116 spheroids when treated at higher H_2_O_2_ concentrations at 24 h is due to spheroid disaggregation because of the cytotoxic effects of H_2_O_2_. Scale bar = 500 µm. **d** Summary of changes in Image-iT™ Red fluorescence intensity of (i) HCT116 (*n* = 3 repeat experiments) and (ii) HN5 (*n* = 2 repeat experiments) spheroids compared to controls. Data are mean ± SD from 14 to 16 spheroids per condition for each experiment. *p < 0.05, **p < 0.01, ns non-significant, Student’s paired *t* test.

Pimonidazole adduct formation confirmed the presence of hypoxia in HCT116 and HN5 spheroid models (Fig. 1a). The reversible live hypoxia marker, Image-iT™ Red, fluoresces at O_2_ concentrations <5% enabling real-time monitoring of changes in oxygenation [18]. A dose-dependent reduction in Image-iT™ Red fluorescence was observed in HCT116 and HN5 spheroids 1 h after treatment with H_2_O_2_, which was significant at [H_2_O_2_] ≥1.2 mM, and consistent with spheroid reoxygenation (Fig. 1b-d, Supplementary Fig. S3). Whilst this reduction in hypoxia was sustained in the HCT116 spheroids up to 6 h post-treatment ( ≥ 2.4 mM H_2_O_2_), red fluorescence reappeared in both the models at the later timepoint of 24 h, suggesting re-emergence of hypoxia (Fig. 1d). Spheroid reoxygenation was further corroborated by staining fixed HCT116 and HN5 spheroids for pimonidazole adducts 24 h following treatment with H_2_O_2_. This effect was observed at [H_2_O_2_] > 1.2 mM (Supplementary Figs. S4 and S5). Collectively these results indicate that the reoxygenation effect is likely to be transient at lower doses and is both dose- and time-dependent.

### Ultrasound imaging confirms sustained release of O_2_ post-intratumoural KORTUC injection

**Fig. 2:**
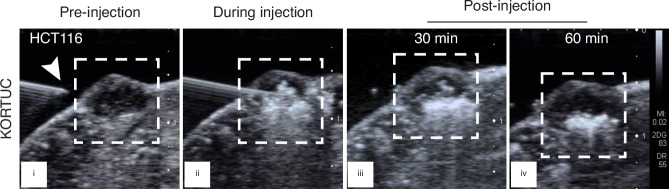
Ultrasound guided intratumoural administration of KORTUC in HCT116 tumour xenografts demonstrating sustained presence of O_2_ microbubbles. US images of an HCT116 tumour (i) pre-injection, (ii) during injection, and (iii) 30 min and (iv) 60 min post-injection of KORTUC. Oxygen microbubbles were seen as a persistent white haze in images (ii)–(iv). The white arrow indicates the needle entry point and the dashed box delineates the tumour region. Reverberation apparent below the needle is an US imaging artefact [36, 37].

During US-guided intratumoural injection in patients, there is immediate formation of oxygen microbubbles (due to the breakdown of H_2_O_2_ into water and oxygen) that disperses throughout the tumour. In the clinical setting, radiotherapy is delivered approximately 1 h following intratumoural KORTUC injection. Using the US-guided injection platform, the spontaneous formation of oxygen microbubbles was visualised immediately following intratumoural injection of KORTUC in both xenograft models (Fig. 2 and Supplementary Fig. S6). On serial US imaging of HCT116 tumours, the echogenicity of the oxygen microbubbles gradually reduced, but persisted for up to 1 h post-injection (Fig. 2). US images acquired during and immediately post-intratumoural injection of sodium hyaluronate revealed no microbubble formation (Supplementary Fig. S7). This confirmed that intratumoural H_2_O_2,_ but not sodium hyaluronate, results in sustained release of oxygen microbubbles, present for at least 1 h post-injection.

### Intratumoural administration of KORTUC significantly decreases tumour hypoxia in HCT116 and HN5 xenografts

**Fig. 3:**
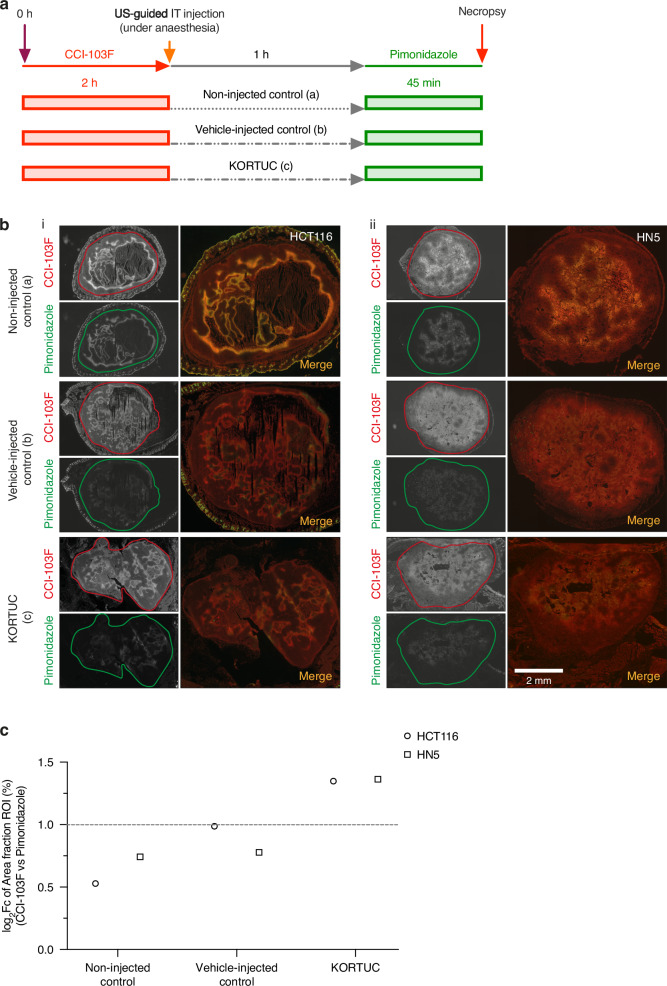
Dual hypoxia marker staining to measure hydrogen peroxide-induced changes in tumour hypoxia in HCT116 and HN5 xenografts. **a** Experimental schema for dual hypoxia staining. **b** Representative immunofluorescence images of CCI-103F (red, pre-treatment) and pimonidazole (green, post-treatment) adduct formation obtained from (a) non-injected control, (b) vehicle-injected control and (c) KORTUC (H_2_O_2_ challenged) (i) HCT116 (*n* = 12) and (ii) HN5 (*n* = 23) tumours, and merged images showing overlapping regions of hypoxia (yellow composite). Whole tumour ROIs used for quantitation are outlined in solid red or green lines. **c** Summary of log_2_Fc determined for percent area of fraction within ROI staining for CCI-103F and pimonidazole adduct formation in HCT116 and HN5 tumours for each treatment regime.

Quantification of CCI-103F and pimonidazole adduct formation (% staining of tumour) demonstrated that intratumoural administration of KORTUC resulted in a significant reduction in tumour hypoxia (>1 log_2_Fc between % CCI-103 staining and % pimonidazole staining) in both xenograft models, in comparison to tumours treated with vehicle (sodium hyaluronate) or non-injected controls (<1 log_2_Fc). Tumours injected with sodium hyaluronate exhibited a non-significant reduction in percentage tumour hypoxia. The merged fluorescence images revealed that all non-injected control tumours demonstrated a high degree of overlap of CCI-103F and pimonidazole adducts, indicating no change in hypoxia (Fig. 3, Supplementary Figs. S8 and 9).

**Fig. 4:**
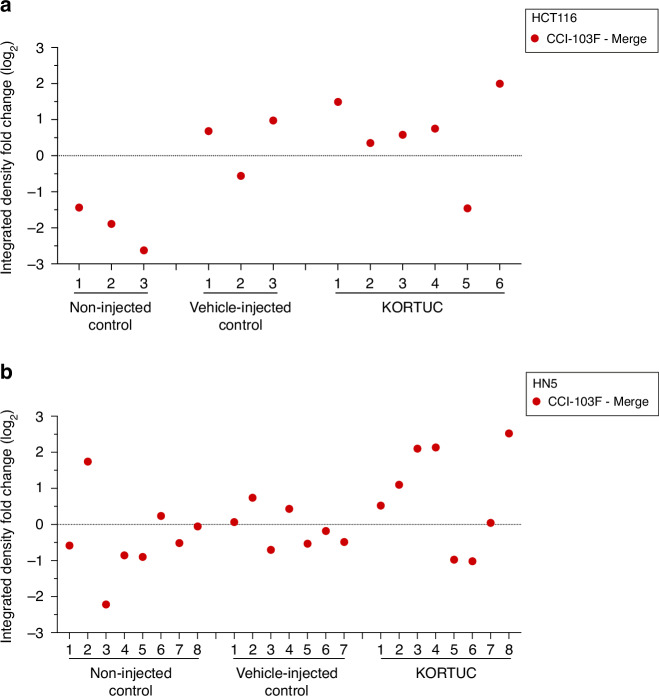
Intratumoural administration of KORTUC decreases tumour hypoxia in both HCT116 and HN5 xenograft models. log_2_Fc showing the difference in integrated density observed in regions stained by CCI-103F to that of the overlapping regions, showing the extent of reoxygenation for the indicated regime for each xenograft (**a**) HCT116 and (**b**) HN5 (Supplementary Figs. S1 and S8–11).

Five of the six HCT116 tumours injected with KORTUC exhibited a marked mismatch between baseline (CCI-103F) and post-injection hypoxia (pimonidazole) (Supplementary Fig. S8). All the non-injected HCT116 xenografts demonstrated a negative log2Fc, whereas in sodium hyaluronate-treated tumours, none had a log2Fc > 1. In the KORTUC-treated HCT116 cohort, 5/6 mice had a positive log2Fc, with 2/6 exhibiting a log2Fc > 1 (Fig. 4a).

In HN5 xenografts there was decreased pimonidazole adduct formation relative to CCI-103F in all treatment groups, but the difference was more marked in the KORTUC cohort (Supplementary Fig. S9 and Fig. 4b). Furthermore, log_2_Fc analysis showed that 4/8 KORTUC-injected HN5 tumours exhibited a log2Fc > 1. There was no evidence of reoxygenation (log2Fc < 1) in the sodium hyaluronate group or in the non-injected group (Fig. 4b). These data demonstrate reoxygenation at the tissue level 1 h after intratumoural KORTUC administration in two xenograft models in vivo.

### KORTUC treatment leads to increased phospho-ATM foci indicative of reoxygenation and increased ROS

**Fig. 5:**
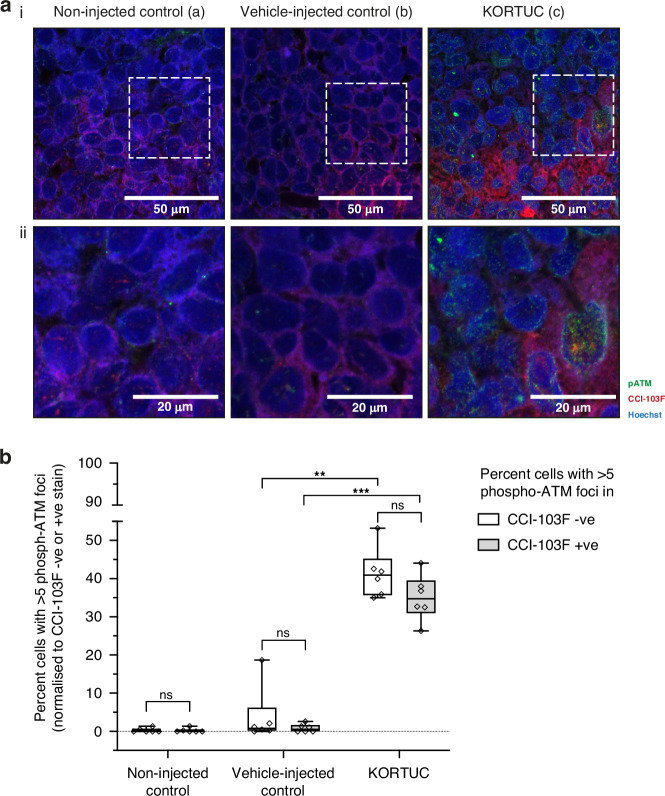
Intratumoral administration of KORTUC increases phospho-ATM foci in HCT116 xenograft indicating increased levels of ROS and reoxygenation. **a** Representative immunofluorescence images showing phospho-ATM (green) and CCI-103F (baseline hypoxia marker, red) in (i) HCT116 xenografts with (**a**) non-injected control or (**b**) vehicle-injected control (sodium hyaluronate) or (**c**) KORTUC. (ii) inset showing zoomed region of interest from (i). **b** Box and Whisker showing the percent increase in cells with >5 phospho-ATM normalized to their CCI-103F staining status for the indicated treatment conditions. Data was obtained by scoring at least 50-100 cells per field of view from at least 5 high power fields (63x) from 6 randomly selected regions in one xenograft per condition (Supplementary Fig. S2). Phospho-ATM foci numbers were compared between the total number of cells in field of view and CCI-103F stained cells. CCI-103F positive cells reflect regions of baseline hypoxia; phospho-ATM foci in positive cells are likely to result from ROS (directly from H_2_O_2_) and/or reoxygenation-induced ROS. CCI-103F negative cells are non-hypoxic; phospho-ATM foci are likely due to ROS directly arising from H_2_O_2_ breakdown. Scale bar: 50 µm; inset 20 µm.

ATM has a critical role in balancing oxidative stress and has been reported to become activated during reoxygenation of hypoxic regions [19]. To identify regions that either underwent oxidative stress due to ROS alone (CCI-103F negative) or ROS and/or reoxygenation-induced ROS (CCI-103F positive), phospho-ATM foci were scored (selected by mapping regions outside the merged dual hypoxia marker) in HCT116 xenografts (Fig. 5, Supplementary Fig. S2). No apparent increase in percentage phospho-ATM foci was observed among the controls (non-injected or vehicle-injected) in both CCI-103F positive and negative cells. In the KORTUC-injected xenografts there were significantly more cells with >5 phospho-ATM foci compared to the control groups. 41% of the CCI-103F negative and 35% of CCI-103F positive stained cells had >5 phospho-ATM foci (Fig. 5b). We postulate that increased phospho-ATM expression in CCI-103F positive cells is likely due to a combination of ROS and/or reoxygenation-induced ROS, whereas in CCI-103F negative cells this is likely due to ROS alone.

## Discussion

The aim of this study was to test the hypothesis that intratumoural administration of KORTUC results in abrogation of tumour hypoxia. The results confirm that intratumoural H_2_O_2_ leads to a reduction in tumour hypoxia, both in 3D spheroid and xenograft models arising from two different cancer cell lines. In vivo, we have demonstrated that this reduction in hypoxia is maintained for up to 1 h post-injection. This mechanism of action can be exploited to overcome hypoxia-induced radiation resistance in a clinically meaningful way, which coincides with the delivery of radiotherapy in the ongoing Phase II clinical trial (NCT03946202) [20]. This study also demonstrates that intratumoural KORTUC administration results in activation of ATM, indicating increased levels of ROS, in addition to reoxygenation.

Image-iT™ Red has been validated for use in 3D spheroids and organoids to visualise and quantify real-time hypoxia [21, 22]. Herein Image-iT™ Red was used to corroborate the results obtained from pimonidazole staining of spheroids at a fixed timepoint and confirm the dynamic reversal of hypoxia over successive time-points in response to H_2_O_2_. The dual hypoxia marker technique (CCI-103F and pimonidazole) has been used in several pre-clinical studies to assess changes in tumour hypoxia [16, 23–25]. By employing this technique we have demonstrated that acute tumour reoxygenation occurs at the tissue level following administration of KORTUC and is sustained for at least 1 h. This highlights that the timing and scheduling of KORTUC relative to RT is important, with regard to maximising radiosensitisation of hypoxic regions. The other implication is that intratumoural KORTUC may be more effective towards the latter part of a course of radiotherapy, when the relative proportion of hypoxic cells within a tumour is expected to be greater.

Anecdotal observations have indicated that reoxygenation is one of several mechanisms that contributes to enhanced cytotoxicity when intratumoural H_2_O_2_ is combined with radiotherapy. A rapid and dramatic increase in oxygen tension of a murine SCCVII tumour following injection of 0.5% H_2_O_2_ in PBS was reported using an invasive polarographic electrode [26]. Takaoka et al. also showed reduced pimonidazole adduct formation following H_2_O_2_ injection in a SCCVII mouse tumour model compared to a non-injected control [27]. However, as these were single images from two different tumours with no baseline measurement of hypoxia, they are not directly comparable due to intertumoural variation. The dual hypoxia marker strategy used herein enabled an assessment of baseline hypoxia in each tumour, thereby providing an internal control.

Interestingly, a few sodium hyaluronate injected control tumours in both models demonstrated a non-significant trend towards reoxygenation, despite there being no release of oxygen microbubbles visualised on US. This was more apparent in the HCT116 model. Sodium hyaluronate is known to bind to CD44, a glycoprotein receptor which is upregulated in hypoxia [28–30]. Such binding may increase vascular permeability and hence oxygen diffusion to hypoxic tumour areas. However, this effect is unlikely to occur in such an acute timeframe. It is, however, reassuring that there is no abrogation of the reoxygenation effect with sodium hyaluronate due to the potential increase in intratumoural pressure. Pre-clinical animal experiments have confirmed that repeated intratumoural injections (on alternate days) of sodium hyaluronate alone have no effect on tumour growth compared to saline controls [27]. The effects of sodium hyaluronate alone are therefore deemed of secondary importance, as in the clinical setting, it is not feasible to inject H_2_O_2_ alone without sodium hyaluronate, due to the rapid breakdown of the compound and increased pain upon injection [5].

The role of KORTUC in reoxygenation and reoxygenation-induced ROS is largely unknown. We wanted to investigate changes in phospho-ATM expression as a molecular marker for the combined effect of ROS and hypoxia-reoxygenation following KORTUC injection. High levels of oxidative stress-induced DNA damage can be detected using γH2AX nuclear foci formation, but under hypoxia γH2AX staining is pan-nuclear due to replication stress in hypoxic cells [31]. During hypoxia-reoxygenation, ATM is phosphorylated (s1981) activating its downstream targets γH2AX, CHK2 and P53 [19, 32]. In response to H_2_O_2_, ATM undergoes oxidation to form ROS-dependent disulphide-linked dimers leading to its phosphorylation at s1981 and other cellular antioxidant responses [33, 34]. We observed a significant increase in phospho-ATM expression following intratumoural administration of KORTUC, suggesting increased oxidative stress in addition to hypoxia-reoxygenation. The increased phospho-ATM observed in CCI-103F positive cells (in regions of reoxygenation, selected by mapping on to areas outside the merged dual hypoxia marker staining in adjacent slides) is likely due to a combination of H_2_O_2_-induced ROS and reoxygenation-induced ROS. Increased phospho-ATM foci in cells negative for CCI-103F (non-hypoxic at baseline) is likely due to H_2_O_2_-induced ROS. These findings support the hypothesis that modulation of intracellular ROS and oxidative stress is another mechanism of action of KORTUC.

Several clinical case series using KORTUC in combination with radiotherapy have been published, the largest of which included 210 patients [35]. A systematically conducted Phase I trial in locally advanced breast cancers confirmed safety with minimal transient injection site pain as the main toxicity with no systemic side effects [2]. Our study suggests that this is a simple and effective method of increasing oxygen delivery directly in tumours, thereby potentially overcoming hypoxia-induced radioresistance. In terms of clinical application, if the ongoing randomised controlled trial in breast cancer (KORTUC Phase II) [20] confirms efficacy, it has the potential for widespread applicability in other tumour types where radiotherapy is the mainstay of curative treatment and hypoxia is known to be a poor prognostic and predictive biomarker.

## Supplementary information

Supplementary Figures

## Data Availability

Data is provided within the manuscript and supplementary information files.
