# Supplementary material for: Tumour reoxygenation after intratumoural hydrogen peroxide (KORTUC) injection: a novel approach to enhance radiosensitivity
Source: BJC Rep. 2024 Oct 8;2:78. doi: 10.1038/s44276-024-00098-y (PMC11461272; doi:10.1038/s44276-024-00098-y)
Supplement: Supplementary file 1 — Supplementary Figures [file 44276_2024_98_MOESM1_ESM.pdf]

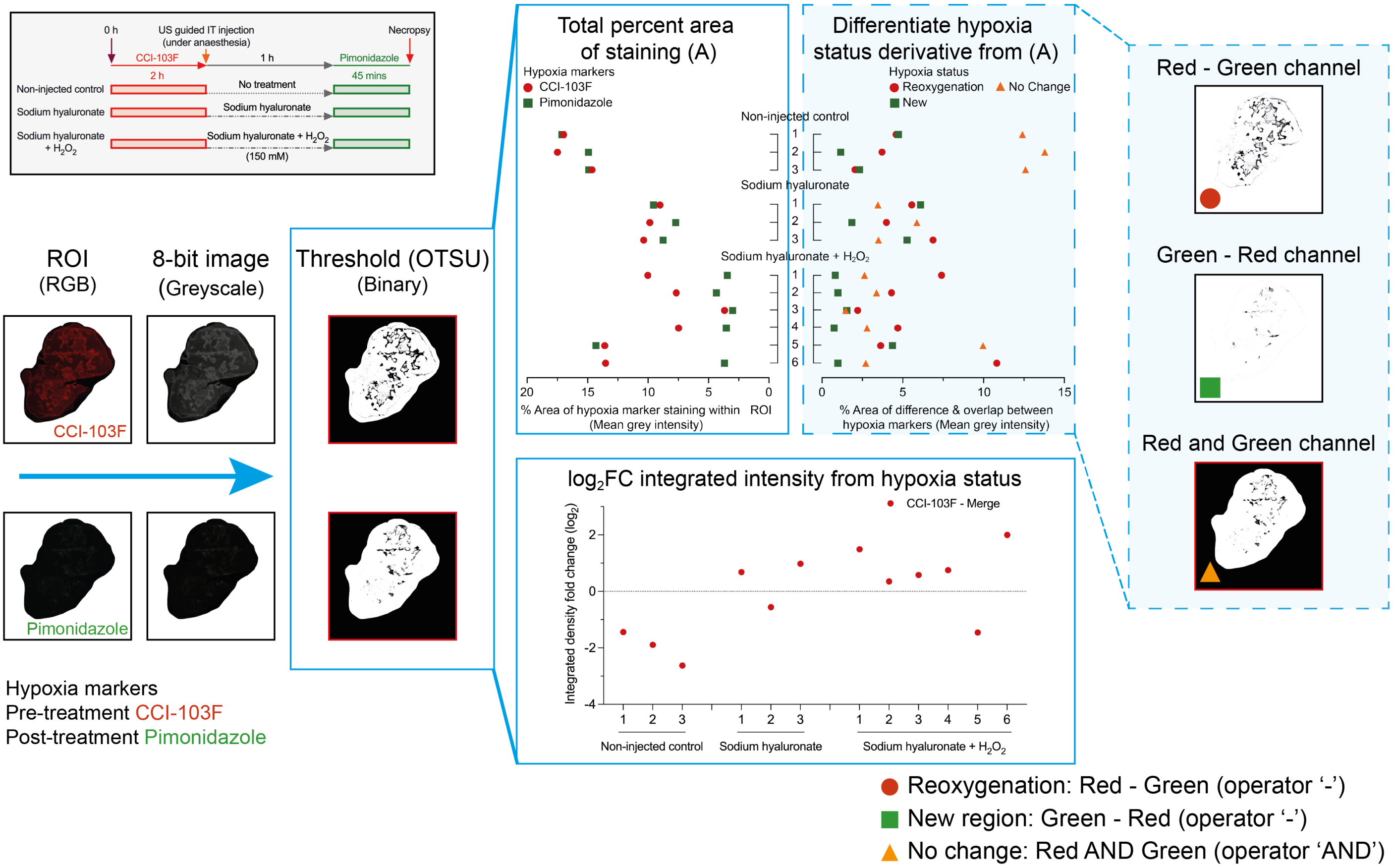

Figure S1. Schema demonstrating image analysis using ImageJ to determine regions of reoxygenation

Region of interest (ROI) was outlined using Adobe photoshop (v23.05) and saved images (jpeg) were processed further using ImageJ. Initially images were converted into greyscale, OTSU threshold applied (manual processing) and the resultant binary output was quantified. The data was plotted for its area and log_2FC integrated intensity (box with solid blue line). Image arithmetic was implemented, subtracting CCI-103F with Pimonidazole (reoxygenation), Pimonidazole with CCI-103F (new regions of hypoxia) and use of 'AND' for CCI-103F overlap with Pimonidazole staining (no change). This method was used to identify the hypoxia status (box with dotted blue line)

Supplementary Figure 2

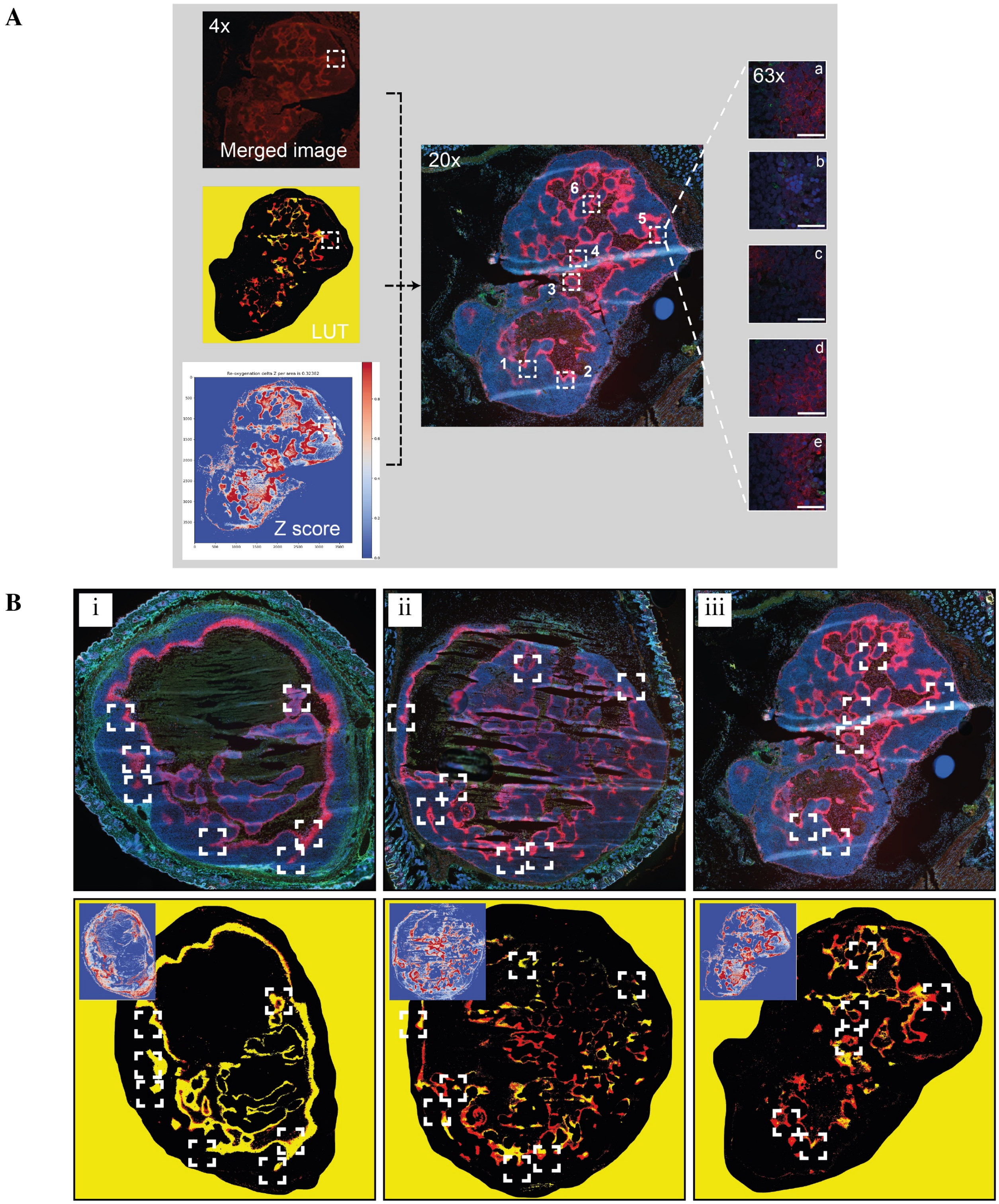

Figure S2. Scheme demonstrating method implemented to determine phospho-ATM expression post intratumoural KORTUC administration in HCT116 xenograft

Scheme (**A**) Matched regions were identified for regions of reoxygenation between the merged dual stained scanned, OTSU threshold and Z score image obtained by calculating ∑ (zScore_CCI-103F - zScore_Pimonidazole) / area of ROI). Once identified this ROI was located on the slide stained for CCI-103F (baseline hypoxia, red), phospho-ATM (green) and Hoechst (blue) and at least 5 high power images were acquired. Using ImageJ counted total number of cells for phospho-ATM foci (at least 1 or >5) also expressed in cells positive for CCI-103F from (A). (**B**) Top panel display phospho-ATM stained images and the bottom panel OTSU threshold applied hypoxia dual staining from Fig. 3Bi and S8, inset zScore images. White dotted boxes, ROI counted for phospho-ATM expression Fig. 5. (i) Non-injected control (ii) Vehicle-injected control (iii) KORTUC.

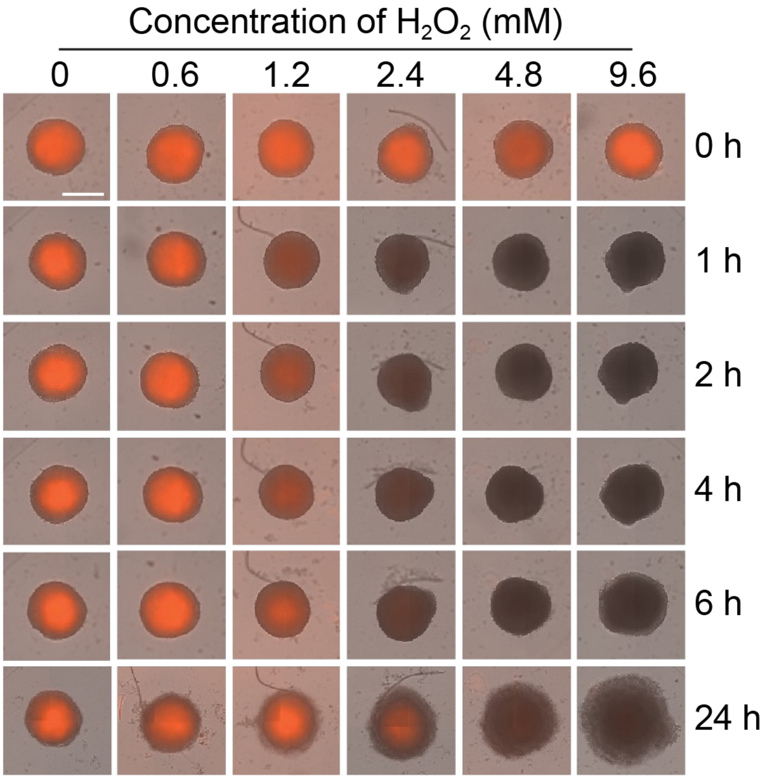

Figure S3. Tumour spheroid reoxygenation following treatment with hydrogen peroxide
Merged brightfield and fluorescence images of representative HCT116 spheroids at various timepoints following H_2O_2 treatment at given concentrations. Reduced fluorescence intensity is indicative of reduction in hypoxia within the spheroids. This was observed in all spheroids at 1h post-treatment with H_2O_2 following concentrations $\geq$ 1.2 mM and was maintained at 24h post-treatment at concentrations $\geq$ 4.8 mM. Scale bar = 500 μm.

A

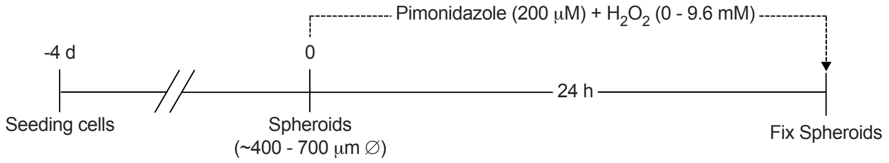

B

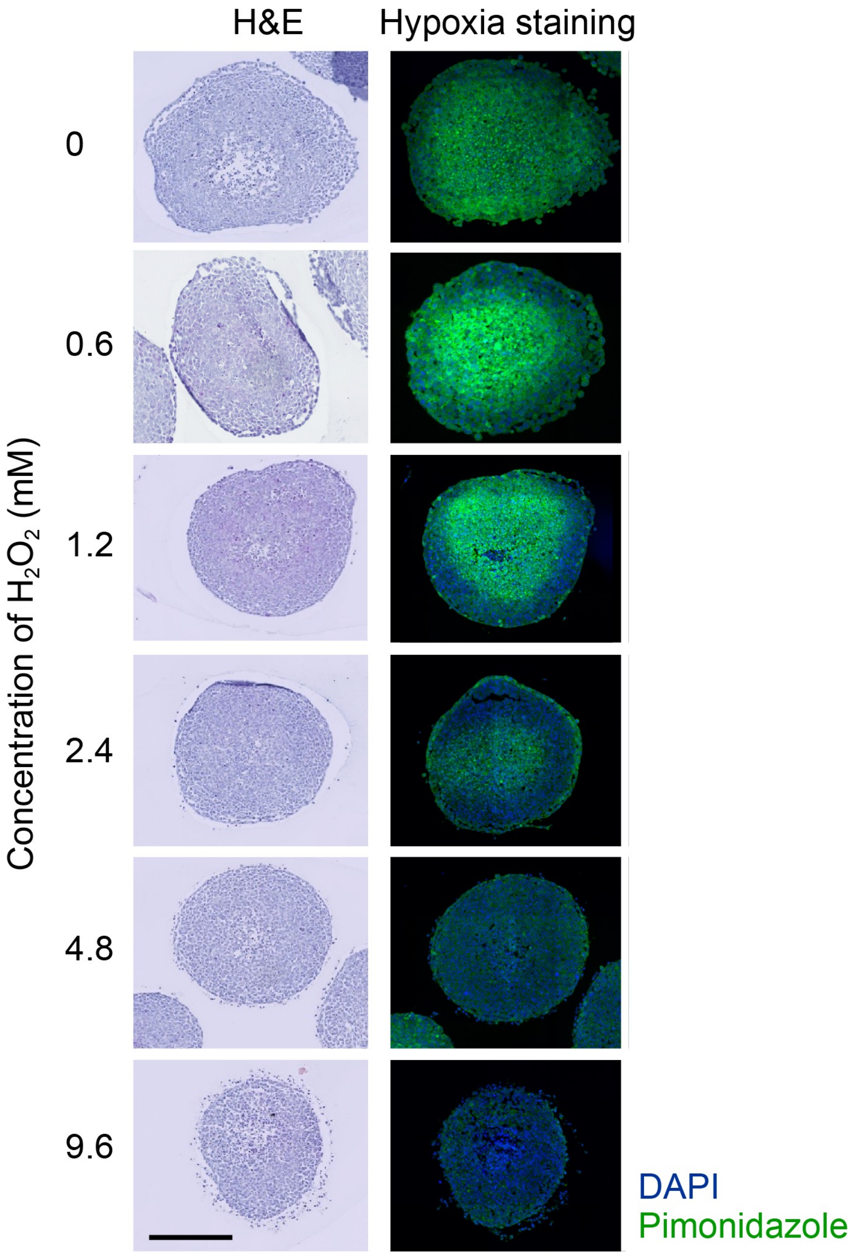

Figure S4. Reduced pimonidazole uptake in HCT116 spheroids treated with hydrogen peroxide

HCT116 spheroids were treated simultaneously with 200 µM pimonidazole and with varying concentrations of H_2O_2 for 24 h before fixation. Spheroid sections were stained with haematoxylin and eosin (left) to visualise morphology or anti-pimonidazole antibodies and DAPI (right) to visualise hypoxia (green) and nuclei (blue). Representative images from 3 to 11 spheroids per condition are shown; Scale bar = 250 µm.

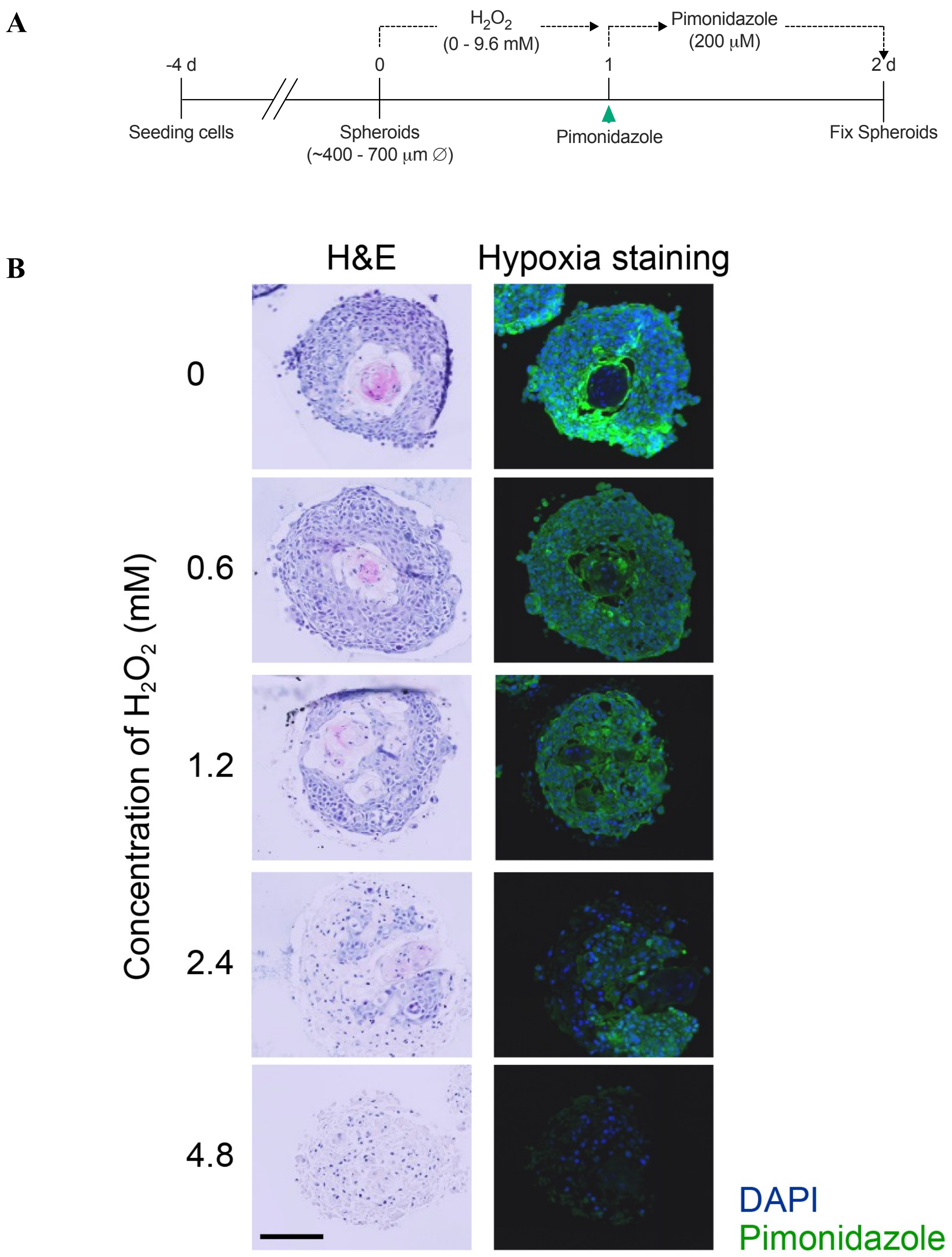

Figure S5. Reduced pimonidazole uptake in HN5 spheroids treated with hydrogen peroxide
HN5 spheroids were treated with varying concentrations of H_2O_2 for 24 h and then with 200 µM pimonidazole for a further 24 h before fixation. Spheroid sections were stained with haematoxylin and eosin (left) to visualise morphology or anti-pimonidazole antibodies and DAPI (right) to visualise hypoxia (green) and nuclei (blue). Representative images from 4 to 15 spheroids per condition are shown; Scale bar = 250 µm.

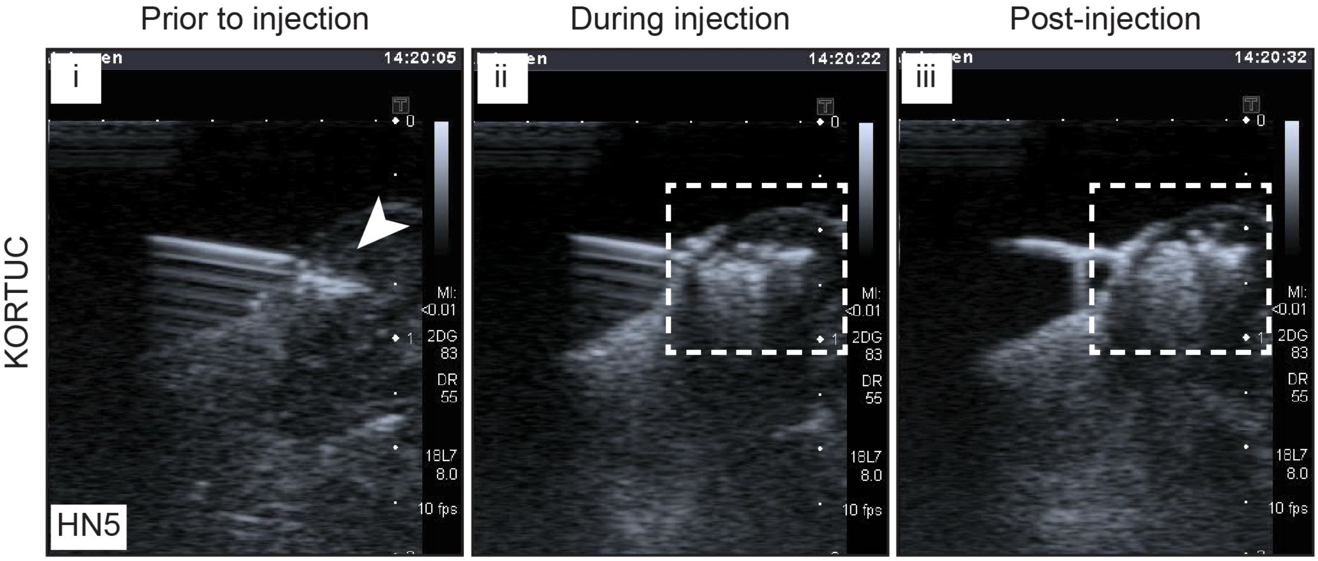

Figure S6. Ultrasound guided intratumoural administration of KORTUC in HN5 xenograft demonstrating release of O_2 microbubbles

Time lapse acquisition of US B-mode contrast images from HN5 tumour in right flank administered intratumoral KORTUC injection (time lapse start 14:20:05 and end time 14:20:32), demonstrating decomposition of H_2O_2 post intratumoural injection. White haze results from release of O_2 microbubbles (i) prior to injection (ii) during and (iii) immediately post-injection. The reverberation observed below the needle is an US imaging artefact (Reusz *et al.*, 2014, Br J Anaesth; Chapman *et al.*, 2006, Anaesthesia). Images acquired at 10 fps. Grey scale bar shown.

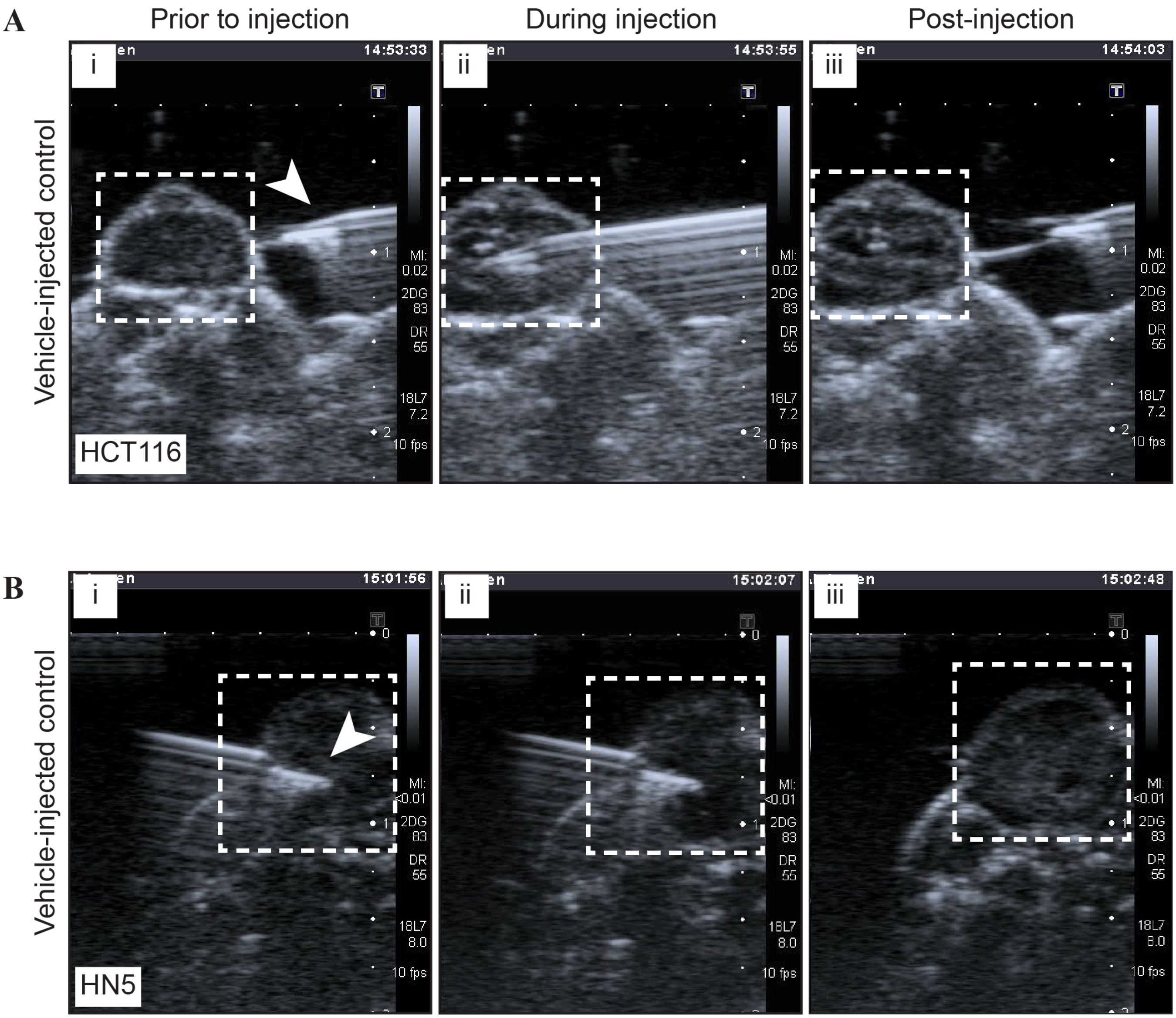

Figure S7 Ultrasound guided intratumoural administration of sodium hyaluronate in HCT116 and HN5 xenograft demonstrating no apparent release of O_2 microbubbles

Time lapse acquisition of US B-mode contrast images from (**A**) HCT116 (time lapse acquisition start 14:53:33 and end time 14:54:03) (**B**) HN5 (time lapse acquisition start 15:01:56 and end time 15:02:48) tumour in right flank administered intratumoral sodium hyaluronate (vehicle-injected control) demonstrating absence of white haze indicative of absence of O_2 microbubbles (i) prior to injection (ii) during and (iii) post-injection. White arrow indicating the entry of needle. The reverberation observed below the needle is an US imaging artefact (Reusz *et al*., 2014, Br J Anaesth; Chapman *et al*., 2006, Anaesthesia). Images acquired at 10 fps. Grey scale bar shown.

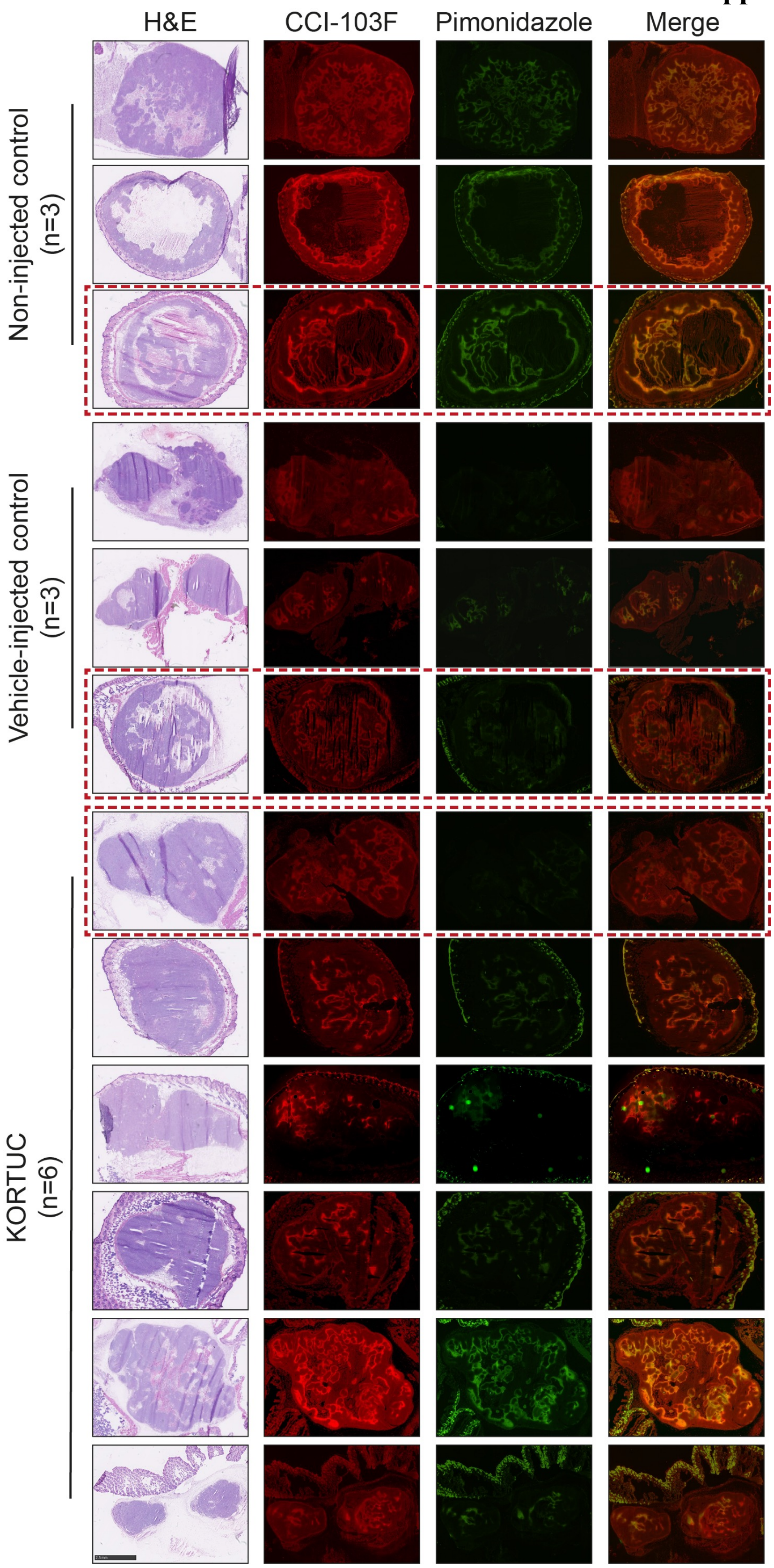

Figure S8. Dual hypoxia staining in HCT116 xenografts

HCT116 xenograft stained for H&E, hypoxia markers CCI-103F (red, baseline pre-treatment hypoxia) and Pimonidazole (green, post-treatment hypoxia) are shown along with their respective composite image (yellow denotes area of overlap between the 2 markers) indicating regions of hypoxia in non-injected controls (n=3), vehicle-injected control (sodium hyaluronate) (n=3) and KORTUC treated xenografts (n=6). Scale bar: 2.5 mm. Solid red dashed line indicates the tumour represented in Figure 3Bi and for analysis of phospho-ATM foci in Figure 5.

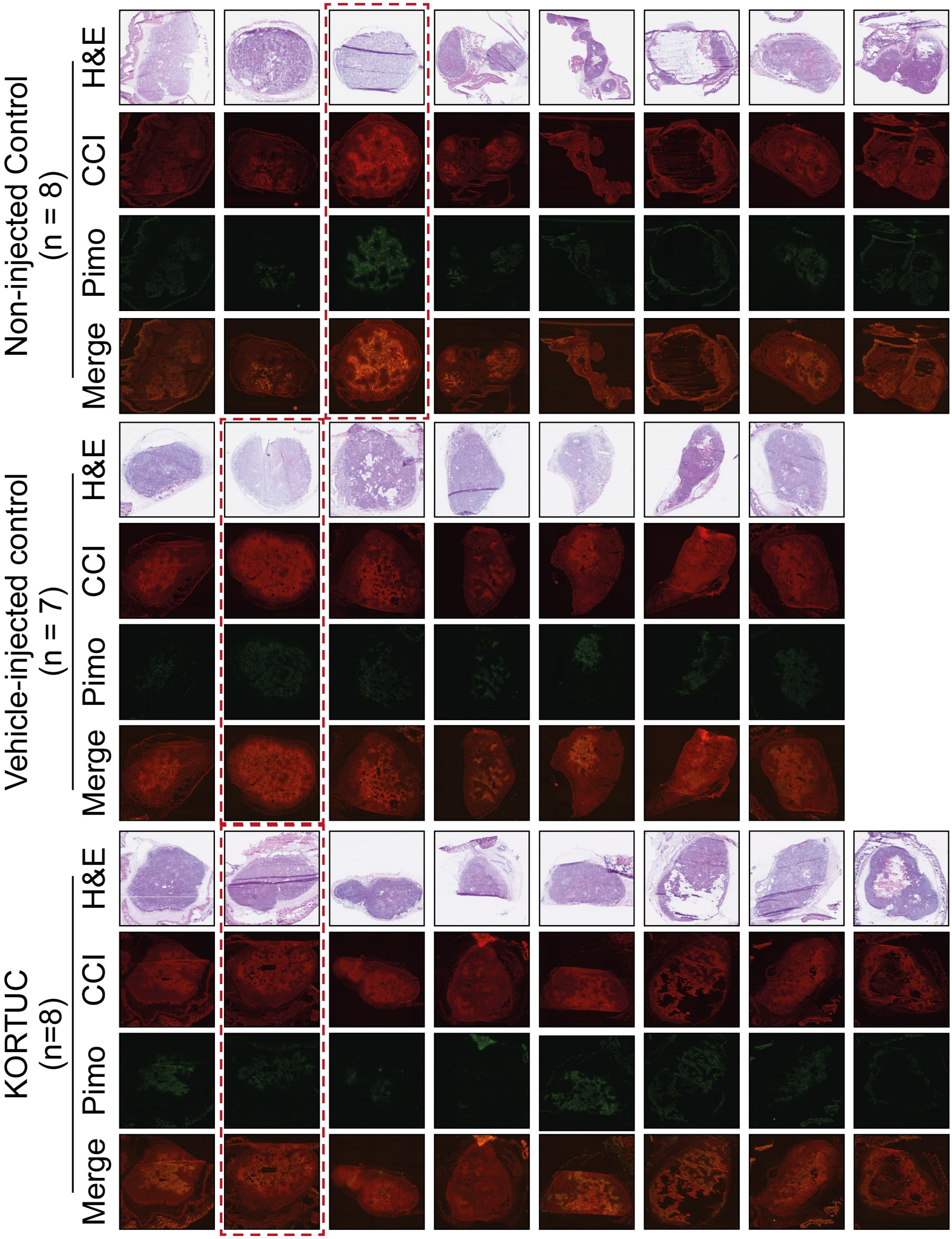

Figure S9. Dual hypoxia staining in HN5 xenografts

HN5 xenografts stained for H&E, hypoxia markers CCI-103F (red, baseline hypoxia) and Pimonidazole (green, post-treatment hypoxia) are shown along with their respective composite image (yellow denotes area of overlap between the 2 markers) indicating regions of hypoxia in non-injected controls (n=8), vehicle-injected control (sodium hyaluronate) (n=7) and KORTUC treated xenografts (n=8). Solid red dashed line indicates the tumour represented in Figure 3Bii.

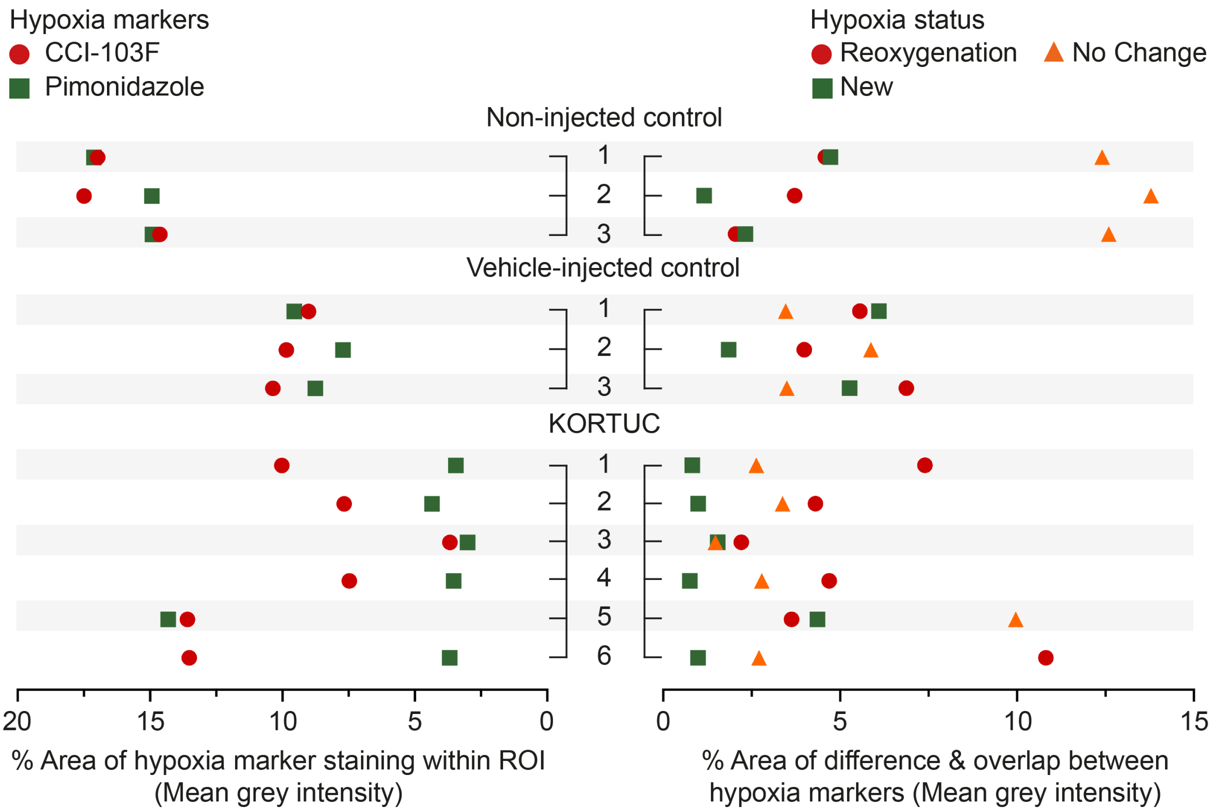

Figure S10. Intratumoural KORTUC administration leads to reduction in tumour hypoxia in HCT116 xenografts

Regions stained with dual hypoxia markers in each individual HCT116 xenograft tumour (n=12) were quantified using ImageJ (Fig. 3, Supplementary Fig. S1 and S8). Left panel showing the mean grey intensity of % area of individual hypoxia marker staining, CCI-103F (baseline, red) and pimonidazole (post-treatment, green); right panel showing the % area of difference and overlap observed between the hypoxia markers from left panel (reoxygenation, red circle; new hypoxia, green square; no change, orange triangle). Non-injected tumours show a high degree of overlap (orange triangle) indicating no change in levels of hypoxia, whereas tumours receiving intratumoural KORTUC exhibit reoxygenation within individual tumours (red circle).

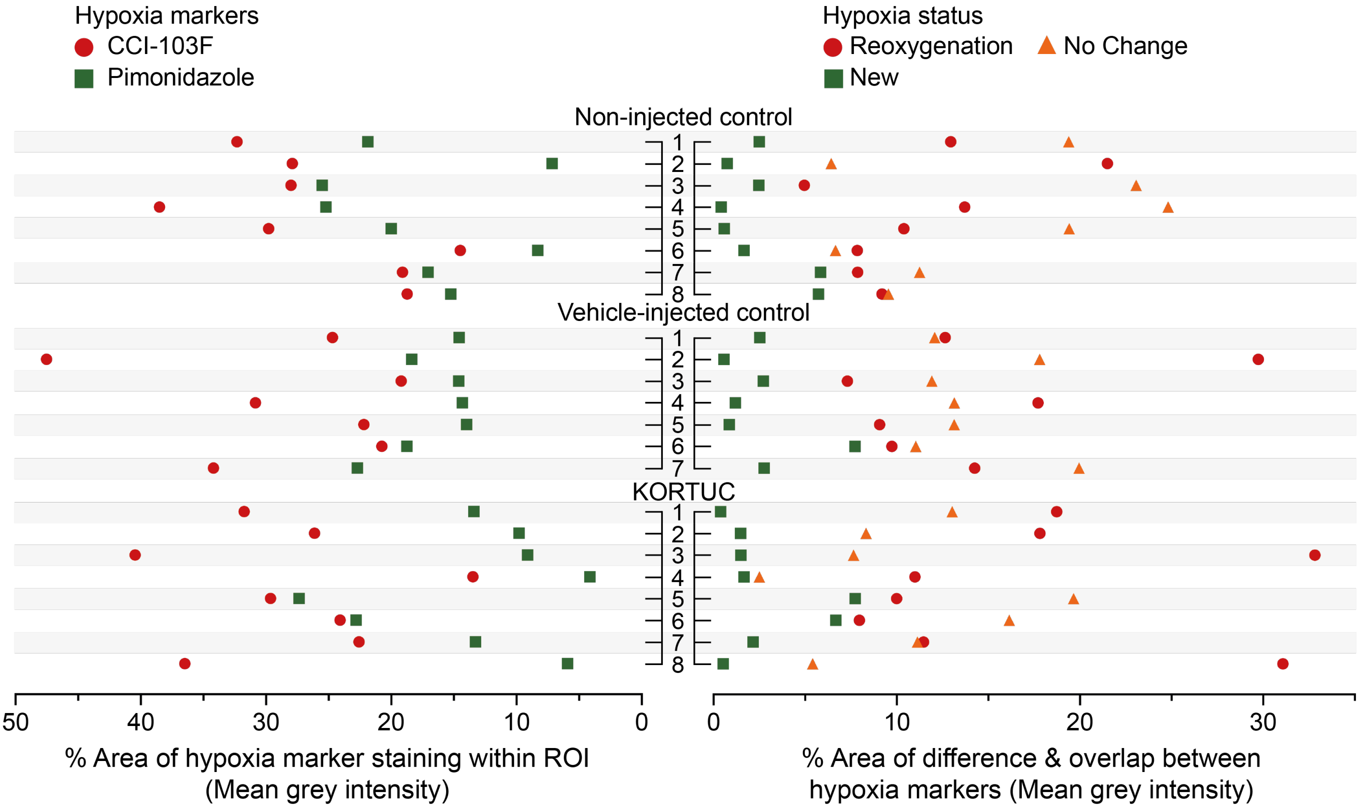

Figure S11. Intratumoural KORTUC administration leads to reduction in tumour hypoxia in HN5 xenografts

Regions stained with dual hypoxia markers in each individual HN5 xenograft tumour (n=23) were quantified using ImageJ (Fig. 3, Supplementary Fig. S1 and S9). Left panel showing the mean grey intensity of % area of individual hypoxia marker staining, CCI-103F (baseline, red) and pimonidazole (post-treatment, green); right panel showing the % area of difference and overlap observed between the hypoxia markers from left panel (reoxygenation, red circle; new hypoxia, green square; no change, orange triangle). Non-injected tumours show a high degree of overlap (orange triangle) indicating no change in levels of hypoxia, whereas tumours receiving intratumoural KORTUC exhibit reoxygenation within individual tumours (red circle).
